# Supplementary material for: The causal relationship between the human gut microbiota and pyogenic arthritis: a Mendelian randomization study
Source: Front Cell Infect Microbiol. 2024 Nov 26;14:1452480. doi: 10.3389/fcimb.2024.1452480 (PMC11629706; doi:10.3389/fcimb.2024.1452480)
Supplement: Supplementary file 1 [file DataSheet1.zip › mendelian test/ebi-a-GCST90027800.csv_leave_one_out.pdf]

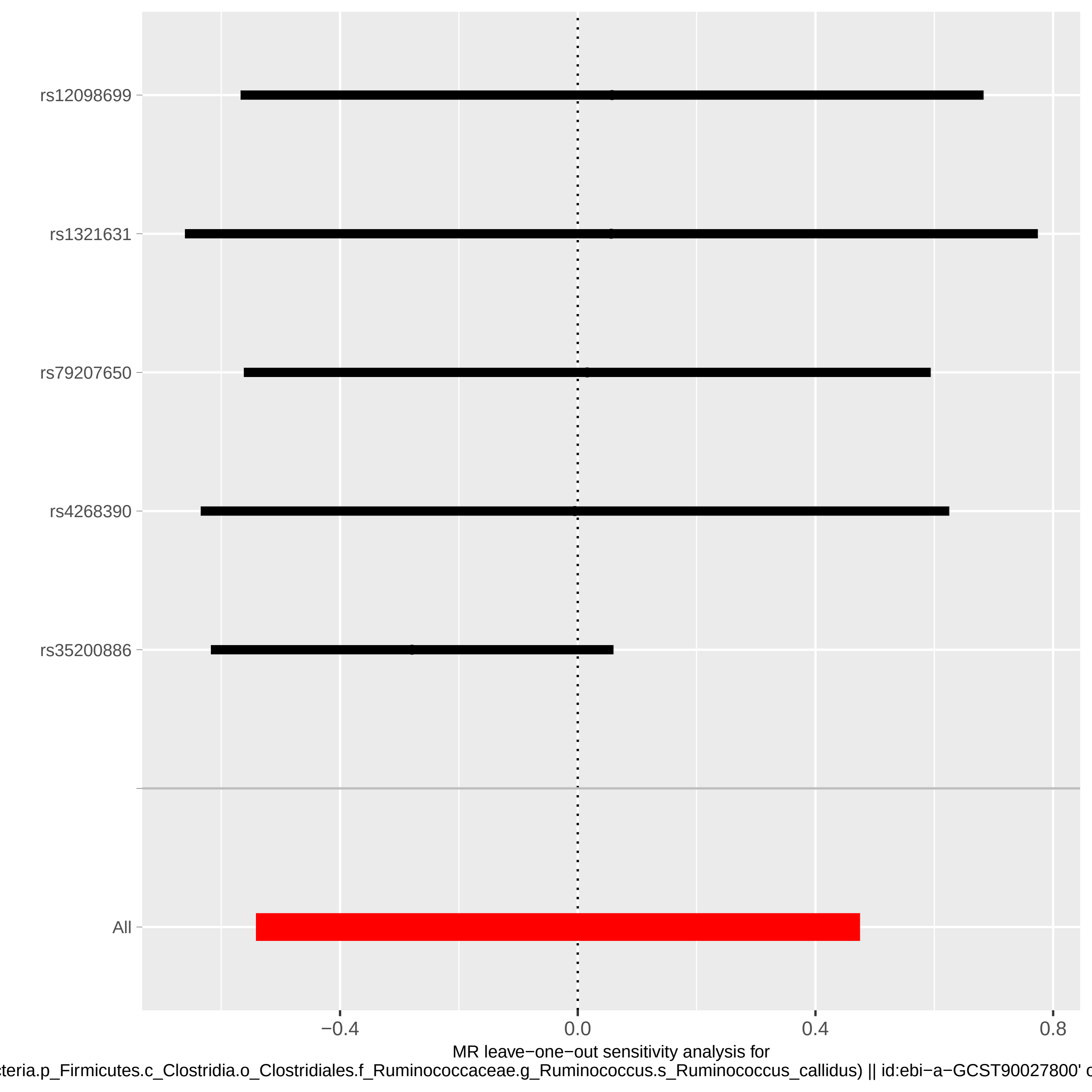

rs12098699
rs1321631
rs79207650
rs4268390
rs35200886
All
−0.4
0.0
0.4
0.8
MR leave−one−out sensitivity analysis for
cteria.p_Firmicutes.c_Clostridia.o_Clostridiales.f_Ruminococcaceae.g_Ruminococcus.s_Ruminococcus_callidus) || id:ebi−a−GCST90027800' o
